# Associations of Mitochondrial Variants With Lipidomic Traits in a Chinese Cohort With Coronary Artery Disease

**DOI:** 10.3389/fgene.2021.630359

**Published:** 2021-03-25

**Authors:** Zixian Wang, Hui Chen, Min Qin, Chen Liu, Qilin Ma, Xiaoping Chen, Ying Zhang, Weihua Lai, Xiaojuan Zhang, Shilong Zhong

**Affiliations:** ^1^Guangdong Provincial People’s Hospital, Guangdong Academy of Medical Sciences, School of Medicine, South China University of Technology, Guangzhou, China; ^2^Department of Pharmacy, Guangdong Provincial People’s Hospital, Guangdong Academy of Medical Sciences, Guangzhou, China; ^3^Guangdong Provincial Key Laboratory of Coronary Heart Disease Prevention, Guangdong Cardiovascular Institute, Guangdong Provincial People’s Hospital, Guangdong Academy of Medical Sciences, Guangzhou, China; ^4^School of Biology and Biological Engineering, South China University of Technology, Guangzhou, China; ^5^Department of Cardiology, The First Affiliated Hospital, Sun Yat-sen University, Guangzhou, China; ^6^Department of Cardiology, Xiangya Hospital, Central South University, Changsha, China; ^7^Department of Clinical Pharmacology, Xiangya Hospital, Central South University, Changsha, China; ^8^Department of Cardiology, Guangdong Provincial People’s Hospital, Guangdong Academy of Medical Sciences, Guangzhou, China

**Keywords:** mitochondrial DNA, polymorphisms, lipidomic, association analyses, coronary artery disease

## Abstract

Plasma lipids have been at the center stage of the prediction and prevention strategies for cardiovascular diseases (CVDs), and novel lipidomic traits have been recognized as reliable biomarkers for CVD risk prediction. The mitochondria serve as energy supply sites for cells and can synthesize a variety of lipids autonomously. Therefore, investigating the relationships between mitochondrial single nucleotide polymorphism (SNPs) and plasma lipidomic traits is meaningful. Here, we enrolled a total of 1,409 Han Chinese patients with coronary artery disease from three centers and performed linear regression analyses on the SNPs of mitochondrial DNA (mtDNA) and lipidomic traits in two independent groups. Sex, age, aspartate aminotransferase, estimated glomerular filtration rate, antihypertensive drugs, hypertension, and diabetes were adjusted. We identified three associations, namely, D-loop_*m*.16089*T*>*C*_ with TG(50:4) NL-16:0, D-loop_*m*.16145*G*>*A*_ with TG(54:5) NL-18:0, and D-loop_*m*.16089*T*>*C*_ with PC(16:0_16:1) at the statistically significant threshold of FDR < 0.05. Then, we explored the relationships between mitochondrial genetic variants and traditional lipids, including triglyceride, total cholesterol (TC), low-density lipoprotein cholesterol (LDLC), and high-density lipoprotein cholesterol. Two significant associations were found, namely *MT-ND6*_*m*.14178*T*>*C*_ with TC and D-loop_*m*.215*A*>*G*_ with LDLC. Furthermore, we performed linear regression analysis to determine on the SNPs of mtDNA and left ventricular ejection fraction (LVEF) and found that the SNP D-loop_*m*.16145*G*>*A*_ was nominally significantly associated with LVEF (*P* = 0.047). Our findings provide insights into the lipidomic context of mtDNA variations and highlight the importance of studying mitochondrial genetic variants related to lipid species.

## Introduction

Coronary artery disease (CAD) is one of the greatest threats to human health (Virani et al., 2020). Lipid metabolism plays a central role in the development of cardiovascular diseases (CVDs) and is considered a therapeutic intervention target for CVDs (Ference et al., 2017; Ouchi et al., 2019; Echouffo-Tcheugui et al., 2020). Lipidomic traits can be used independently in predicting long-term CVD risk and adverse clinical outcome. The application of lipidomic traits for risk prediction is more comprehensive than traditional lipids, namely triglycerides (TRIG), total cholesterol (TC), low-density lipoprotein (LDLC), and high-density lipoprotein cholesterol (HDLC) (Razquin et al., 2018; Cadby et al., 2020; Poss et al., 2020).

Mitochondria serve as energy supply sites for cells and can synthesize a variety of lipids autonomously, such as phosphatidylglycerol, cardiolipin, and phosphatidic acid (Horvath and Daum, 2013). Mitochondrial dysfunction can cause various diseases, including CVDs (cardiomyopathy, heart failure, and arrhythmia) (Marín-García and Goldenthal, 2002; Yang et al., 2014; Wahbi et al., 2015). Human mitochondrial DNA (mtDNA) is in a circular double-stranded state and approximately 16.5 kb in length and encodes 37 genes (Sobenin et al., 2012). The replication of mtDNA starts with the displacement loop (D-loop) and contains a transcript promoter adjacent to the D-loop (Sharma and Sampath, 2019). Although the exact function of the D-loop is unclear, this region has a high sequence variability (Ingman et al., 2000). The sequences outside the D-loop region are all exons and encode genes, and thus mtDNA mutations are more likely to occur in the coding region than autosomal DNA mutations and directly affect the physiological functions of the genes encoded by them. Therefore, identifying the associations between mitochondrial genetic patterns and lipid species can provide a deeper understanding of lipid metabolism and its biological links to clinical features and disease progression.

Here, we performed large-scale association analyses on mtDNA variations and lipidomic traits in a Han Chinese population to obtain insights into the relationships between mitochondrial genetic variants and plasma lipidomic traits in CAD patients. We recruited 1,409 subjects with CAD from three centers and used multiple linear regression to explore the relationships between the 42 single nucleotide polymorphisms (SNPs) of mtDNA and 309 lipid species in two independent groups. Moreover, we investigated the relationships between mtDNA variations and the plasma profiling of traditional lipids, including TRIG, TC, LDLC, and HDLC to search more SNPs affecting these lipids. We then explored the effects of mitochondrial genetic variants on heart function by using linear regression to analyze the variations in mtDNA with left ventricular ejection fraction (LVEF). Our findings may provide novel insights into the lipidomic context of mtDNA variations and provide a potential target for pharmacological intervention and optimized treatment of patients with CAD.

## Materials and Methods

### Study Population

Two groups were studied, and the Group I and Group II were mainly grouped according to the time of enrolment. Subjects of the Group I were sequentially enrolled from Guangdong Provincial People’s Hospital between January 2010 and December 2013. The samples in Group I were obtained from patients who had complete baseline characteristics and genetic data in our previous study (Liu et al., 2019). Group II was a multicenter cohort including patients from three centers (Guangdong Provincial People’s Hospital, Xiangya Hospital, Center South University, and the First Affiliated Hospital, Sun Yat-sen University) from September 2017 to October 2018 to illustrate the repeatability and representation of the results from the Group I. All patients enrolled in this study had taken statin lipid-lowering drugs, diagnosed with CAD through coronary angiography, and had undergone percutaneous coronary intervention (PCI). The exclusion criteria in Group II were the same as those used in the previous study, namely, (1) age < 18 years or >80 years; (2) renal insufficiency [defined as serum creatinine concentration > two times the upper limit of normal (230 μmol/L) and renal transplantation or dialysis]; (3) hepatic insufficiency [defined as serum transaminase concentration > two times the upper limit of normal (80 U/L) or diagnosis of cirrhosis]; (4) pregnancy or lactating status; (5) advanced cancer or hemodialysis; (6) history of thyroid problems and the use of antithyroid drugs or thyroid hormone medication; and (7) incomplete information about cardiovascular events during follow-up.

Our study was approved by the Medical Ethical Review Committee of Guangdong General Hospital (Nos. GDREC2010137 and GDREC2017071H) and conducted in accordance with the Declaration of Helsinki. Informed consent (Nos. 20100910 and 20170211) was obtained each participant enrolled in the study. Baseline information, such as demographics, medical history, biochemical measurements, and medication, were all obtained from the hospital information database.

### Lipidomic Data

The lipidomic data of the two groups were obtained through widely targeted lipidomic profiling. Details were described in our previous study (Qin et al., 2020), see Supplementary Material. In general, 667 lipid species were detected in the Group I, which contained 14 categories, namely, monoglyceride (MG), cholesteryl esters (CE), diacylglycerol (DG), triacylglycerol (TG), phosphatidic acids (PA), phosphatidylcholines (PC), phosphatidylglycerol (PG), phosphatidylserines (PS), phosphatidylethanolamines (PE), lysophosphatidic acids (LPA), lysophosphatidylcholine (LPC), lysophosphatidylethanolamine (LPE), hemolytic serine (LPS), and ceramides (Cer). 687 lipid species were annotated in Group II, and a total of 309 lipid species were detected in both groups. For lipidomic analyses, raw signals with more than half of missing rate in the QC samples (those with zero ion intensity) were removed. Missing lipidomic data were imputed by replacing the missing value with a minimum value of the lipid species quantified. The lipidomic data after the batch effect correction by Quality Control–based Robust LOESS (Locally Estimated Scatterplot Smoothing) Signal Correction (QC-RLSC) algorithm was then scaled by pareto scaling, which utilized the square root of the standard deviation as the scaling factor (van den Berg et al., 2006).

### Mitochondrial Genetic Data

For Group II, each eligible patient fasted for at least 8 h. This procedure was performed to minimize the influence of nutrition on lipid species levels. Blood samples were collected using ethylenediaminetetraacetic acid-coated tubes. Whole blood samples were separated into plasma and hemocytes within 2 h through centrifugation at 2,095 *g* for 10 min at 4°C and then stored at -80°C for further analysis. DNA was extracted from the hemocyte samples for genotyping with a TGuideM16 automatic nucleic acid extractor (Cat. No. OSE-M16) and genomic DNA extraction kit (Cat. No. OSR-M102; TIANGEN). Concentration quantification and electrophoresis were conducted for DNA QC, and qualified DNA was genotyped using an Illumina Infinium global screening array (GSA) bead chip. In brief, the 140 SNPs of mtDNA were genotyped for the two groups. We combined the mitochondrial genetic data of both groups, and the exclusion criteria were as follows: (1) maximum per-person missing rate > 0.05; (2) maximum per-SNP missing rate > 0.05; (3) Hardy–Weinberg disequilibrium *P* value < 1e-6; and (4) minor allele frequency < 0.01.

### Association Analyses

Linear regression analyses were conducted at each qualified SNPs of mtDNA for the levels of 309 lipid species presented in Group I and Group II. Sex, age, aspartate aminotransferase (AST), estimated glomerular filtration rate (eGFR), antihypertensive drugs (including β-blockers, angiotensin converting enzyme inhibitors, calcium channel blockers, and proton pump inhibitors), hypertension, and diabetes mellitus were adjusted. All association analyses were conducted using PLINK v2.0 software (Chang et al., 2015).

### Meta-Analysis

We used an inverse-variance weighted method meta-analysis, which was based on the effect sizes, and the standard errors adjusting for genomic control were used to combine the association results for the two groups. The heterogeneities in each association between the datasets were tested using the Cochran’s Q test. The analyses above were conducted using METAL software (Willer et al., 2010). Only the associations that met the following conditions were presented: (1) *P-*value of both groups < 0.05 and (2) the *P*-value of heterogeneity of effects between the two groups > 0.001. The results of meta-analysis were adjusted to account for multiple testing by the Benjamini-Hochberg false discovery rate (FDR) method and associations were considered statistically significant at FDR < 0.05. All analyses were performed in R v3.6.1^1^.

### Associations of mtDNA With Traditional Lipids and Heart Function

To explore the influence of mitochondrial genetic variants with traditional lipids and heart function, multiple linear regression of the qualified SNPs of mtDNA to the traditional lipids (TRIG, TC, LDLC, and HDLC), and the heart function (LVEF) were conducted with adjusting for confounding factors including sex, age, AST, eGFR, antihypertensive drugs, hypertension, and diabetes mellitus. Benjamini-Hochberg FDR was used in correcting the number of the SNPs of mtDNA for multiple hypothesis testing, and statistical analyses were performed using PLINK v2.0 and R v3.6.1.

## Results

### Patients Characteristics

This study enrolled 1,409 Han Chinese patients with CAD who were undergoing PCI therapy. A total of 914 subjects were included in Group I (62.90 ± 10.06 years old), and 495 subjects from three centers (353 patients from Guangdong Provincial People’s Hospital, 138 patients from Xiangya Hospital, Center South University, and 4 patients from the First Affiliated Hospital, Sun Yat-sen University) were included in Group II (61.98 ± 9.81 years old). All the subjects had complete mitochondrial genetic, lipidomic, and traditional lipids data. The average TRIG levels were 1.61 ± 1.13 and 1.84 ± 1.90 mmol/L for Group I and Group II, respectively. The average TC levels were 4.25 ± 1.09 and 4.28 ± 1.78 for Group I and Group II. The average LDLC levels were 2.56 ± 0.90 and 2.70 ± 0.97 mmol/L for Group I and Group II. The average HDLC levels were 0.96 ± 0.25 and 0.99 ± 0.25 mmol/L for Group I and Group II. The detailed demographic characteristics are presented in Table 1.

**TABLE 1:** Baseline characteristics of the two groups.

Characteristics	Value *N* (%) or mean ± SD	
	Group I	Group II
**Demographic data**		
Size	914	495
Age	62.90 ± 10.06	61.98 ± 9.81
Sex (male)	733 (80.20)	367 (74.14)
BMI, kg/m^2^	24.14 ± 3.10	24.13 ± 3.07
Comorbidities		
Arrhythmia	78 (8.53)	44 (8.89)
Diabetes	251 (27.46)	141 (28.48)
Heart failure	77 (8.42)	221 (44.65)
Hypertension	546 (59.74)	300 (60.61)
Hyperlipidemia	101 (11.05)	66 (13.33)
**Baseline biochemical measurements**		
ALT, U/L	27.54 ± 12.99	25.82 ± 18.71
AST, U/L	26.76 ± 10.77	26.13 ± 39.12
CK, U/L	114.09 ± 115.89	122.87 ± 352.43
eGFR, ml/min/1.73 m^2^	95.85 ± 77.82	95.51 ± 126.21
CKMB, U/L	7.60 ± 6.08	12.58 ± 13.73
TC, mmol/L	4.25 ± 1.09	4.28 ± 1.78
LDLC, mmol/L	2.56 ± 0.90	2.70 ± 0.97
HDLC, mmol/L	0.96 ± 0.25	0.99 ± 0.25
TRIG, mmol/L	1.61 ± 1.13	1.84 ± 1.90
GLUC, mmol/L	6.66 ± 2.64	5.95 ± 2.12
Lpa, mg/L	306.68 ± 325.61	287.03 ± 332.98
APOA, g/L	1.04 ± 0.28	1.17 ± 0.24
BNP, pg/mL	782.52 ± 1658.15	1066.73 ± 3209.70
**Medication**		
β-blockers	810 (88.62)	427 (86.26)
ACEIs	580 (63.46)	239 (48.28)
CCBs	253 (27.68)	150 (30.30)
PPIs	445 (48.69)	346 (69.90)
SYNTAX	16.37 ± 10.71	15.97 ± 13.35

*BMI, body mass index; ALT, alanine aminotransferase; AST, aspartate aminotransferase; CK, creatine kinase; eGFR, estimated glomerular filtration rate; CKMB, creatine kinase MB; TC, total cholesterol; LDLC, low-density lipoprotein cholesterol; HDLC, high-density lipoprotein cholesterol; TRIG, triglyceride; GLUC, glucose; Lpa, lipoprotein (a); APOA, apolipoprotein a; BNP, B-type natriuretic peptide; ACEIs, angiotensin converting enzyme inhibitors; CCBs, calcium channel blockers; PPIs, proton pump inhibitors; SYNTAX, Synergy between PCI with TAXUS and Cardiac Surgery score.*

### Mitochondrial Genetic Variants and Lipidomic Data

As indicated in the Materials and methods section, the 42 SNPs of mtDNA, including nine SNPs in the D-loop region, two SNPs on the 12S rRNA, two SNPs on the 16S rRNA, one SNP on the tRNA, and 28 SNPs in the exon region met the quality control criteria for merged genotype data. In the lipidomic data, 309 plasma lipid species, including 7 CE, 23 DG, 172 TG, 50 PC, 18 LPC, 19 LPE, 1 LPS, and 19 Cer were included for the association analyses conducted on each group.

### Associations of mtDNA Variations With Lipid Species

**FIGURE 1:**
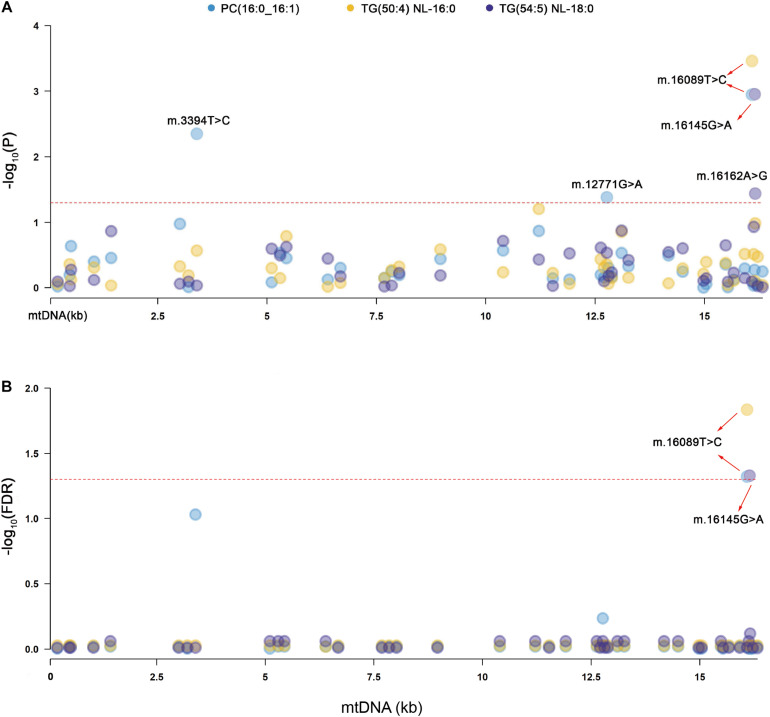
Manhattan plots for the significant associations of mitochondrial genetic variants with lipid species. PC, phosphatidylcholines, TG, triacylglycerol. **(A)** The red dashed line indicates the threshold of nominal significance on lipid species (*P* < 0.05). **(B)** The red dashed line indicates the threshold of statistical significance on lipid species after corrected (FDR < 0.05).

Primary association analyses were conducted using the 42 SNPs of mtDNA with 309 lipid species in the two groups. The association results were merged through meta-analysis. *P-*values in the two groups are below 0.05, and no significant heterogeneity between them was observed (see section “Materials and Methods”; Table 2). In total, we identified seven associations of mitochondrial genetic variants with lipid species (*P* < 0.05). Three associations were statistically significant after FDR correction (Table 2 and Figure 1). m.16089T > C (T > C) in D-loop was associated with the increased level of TG(50:4) NL-16:0 (*P* = 0.00035, FDR = 0.01462), m.16145G > A (A > G) in D-loop was associated with the decreased level of TG(54:5) NL-18:0 (*P* = 0.00111, FDR = 0.04670), and m.16089T > C (T > C) in D-loop was associated with the increased level of PC(16:0_16:1) (*P* = 0.00113, FDR = 0.04759). Four of them were in nominally significant associations (*P* < 0.05, but FDR > 0.05). Specifically, m.15924A > G (A > G) in *tRNA-Thr* was nominally significantly associated with the increased level of TG(58:9) NL-20:4 (*P* = 0.00256, FDR = 0.10756), m.16217T > C (T > C) in D-loop was nominally significantly associated with the increased levels of Cer(m18:1/26:0) (*P* = 0.00401, FDR = 0.16859), and TG(58:3) NL-18:2 (*P* = 0.00567, FDR = 0.23801), and m.6680T > C (T > C) in *MT-CO1* was nominally significantly associated with the increased level of CE(18:1) (*P* = 0.00749, FDR = 0.20076).

**TABLE 2:** Significant associations of mitochondrial genetic variants with lipid species.

Lipid species	mtSNP	Gene/region	Test	EAF	Effect	SE	*P*-value of meta-analysis	FDR	*P*-value of group I	*P*-value of group II	heterogeneity
TG(50:4) NL-16:0	m.16089T > C	*D-loop*	T > C	0.98	0.65	0.18	0.00035	0.01462	0.01410	0.00929	0.78
TG(54:5) NL-18:0	m.16145G > A	*D-loop*	A > G	0.01	−0.54	0.17	0.00111	0.04670	0.02215	0.00403	0.08
PC(16:0_16:1)	m.16089T > C	*D-loop*	T > C	0.98	0.35	0.11	0.00113	0.04759	0.01040	0.03987	0.62
TG(58:9) NL-20:4	m.15924A > G	*tRNA Thr*	A > G	0.99	0.63	0.21	0.00256	0.10756	0.04615	0.01651	0.41
Cer(m18:1/26:0)	m.16217T > C	*D-loop*	T > C	0.87	0.29	0.10	0.00401	0.16859	0.04969	0.00794	0.10
TG(58:3) NL-18:2	m.16217T > C	*D-loop*	T > C	0.87	0.24	0.09	0.00567	0.23801	0.04579	0.02383	0.22
CE(18:1)	m.6680T > C	*MT-CO1*	T > C	0.91	0.08	0.03	0.00749	0.20076	0.04293	0.04623	0.33

*EAF, effect allele frequency; TG, triacylglycerol; PC, phosphatidylcholines; Cer, ceramides; TG, triacylglycerol; CE, cholesteryl esters; SE, standard error; FDR, false discovery rate.*

### Relationships Between mtDNA Variations and Traditional Lipids

**FIGURE 2:**
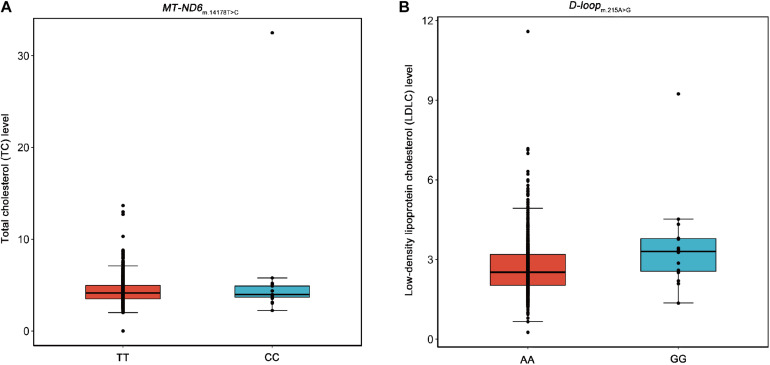
Box plots for the SNPs m.14178T > C and m.215A > G against the levels of TC and LDLC. Five lines from top to bottom for each box were upper extreme, upper quartile, median, lower quartile, and lower extreme. Whiskers were drawn between upper extreme and upper quartile, lower quartile and lower extreme, and the dots above upper extreme or below lower extreme were outliers. **(A)** The SNP m.14178T > C (T > C) in *MT-ND6* against the level of TC. **(B)** The SNP m.215A > G (A > G) in D-loop against the level of LDLC.

Moreover, we performed association analyses between the 42 SNPs of mtDNA and four traditional lipids to explore the relationships of mtDNA variants and traditional lipids (TRIG, TC, LDLC, and HDLC), using a merged genotype and lipids data (*N* = 1409). No SNP was statistically significantly associated with TRIG. The results of potential associations (*P* < 0.05) were presented in Table 3. After FDR corrections, two associations were considered statistically significant (FDR < 0.05). m.14178T > C (T > C) in *MT-ND6* was associated with the increased level of TC, and m.215A > G (A > G) in D-loop was associated with the increased level of LDLC (Figure 2). All 42 SNPs with the four lipids were presented in Supplementary Tables 1–4.

**TABLE 3:** Relationships of mtDNA variations with traditional lipids (*P* < 0.05).

Lipid	mtSNP	Gene/region	Test	Effect	SE	*P*	FDR
TC	m.14178T > C	*MT-ND6*	T > C	1.45	0.34	0.000016	0.000691
	m.215A > G	*D-loop*	A > G	1.08	0.36	0.002684	0.056354
	m.6680T > C	*MT-CO1*	T > C	0.28	0.13	0.026426	0.369958
	m.12811T > C	*MT-ND5*	T > C	0.27	0.13	0.036252	0.380642
	m.3010G > A	*MT-RNR2*	G > A	−0.23	0.12	0.045509	0.382271
LDLC	m.215A > G	*D-loop*	A > G	0.80	0.24	0.001098	0.046129
	m.6680T > C	*MT-CO1*	T > C	0.25	0.09	0.002930	0.061529
	m.12811T > C	*MT-ND5*	T > C	0.24	0.09	0.006208	0.086907
	m.3010G > A	*MT-RNR2*	G > A	−0.20	0.08	0.012639	0.132706
	m.7684T > C	*MT-CO2*	T > C	0.19	0.08	0.015906	0.133611
HDLC	m.6680T > C	*MT-CO1*	T > C	0.06	0.02	0.009879	0.203592
	m.12811T > C	*MT-ND5*	T > C	0.06	0.02	0.010075	0.203592
	m.7684T > C	*MT-CO2*	T > C	0.05	0.02	0.014542	0.203592
	m.7853G > A	*MT-CO2*	G > A	0.05	0.02	0.025186	0.245955
	m.11914G > A	*MT-ND4*	G > A	−0.06	0.03	0.029280	0.245955

*TC, total cholesterol; LDLC, low-density lipoprotein cholesterol; HDLC, high-density lipoprotein cholesterol; SE, standard error; FDR, false discovery rate.*

### Relationships Between mtDNA Variations and LVEF

We performed further analyses to explore the relationships between the SNPs of mtDNA and heart function. LVEF is a continuous variable that can be used as an index for evaluating heart function, and a low LVEF value implies poor heart function. A total of 1,151 subjects with LVEF data were available in linear regression analyses, whose LVEF(%) was 59.92 ± 11.47 (mean ± standard deviation). Three SNPs were nominally significantly associated with LVEF (*P* < 0.05 but FDR > 0.05). m.1048C > T (C > T) in *MT-RNR1* was nominally significantly associated with decreased LVEF, m.12705C > T (T > C) in *MT-ND5* was nominally significantly associated with decreased LVEF, and m.16145G > A (G > A) in D-loop was nominally significantly associated with decreased LVEF (Table 4). The SNP m.16145G > A showed a potential association with LVEF (*P* < 0.05) among the SNPs that were statistically significantly associated with lipid species (FDR < 0.05). All 42 associations were presented in Supplementary Table 5.

**TABLE 4:** Relationships of mtDNA variations with LVEF (*P* < 0.05).

mtSNP	Gene/region	Test	Effect	SE	*P*	FDR
m.1048C > T	*MT-RNR1*	C > T	−5.84	2.40	0.01509	0.63390
m.12705C > T	*MT-ND5*	T > C	−1.37	0.66	0.03899	0.65863
m.16145G > A	*D-loop*	G > A	−5.58	2.81	0.04705	0.65863

*SE, standard error; FDR, false discovery rate.*

## Discussion

**FIGURE 3:**
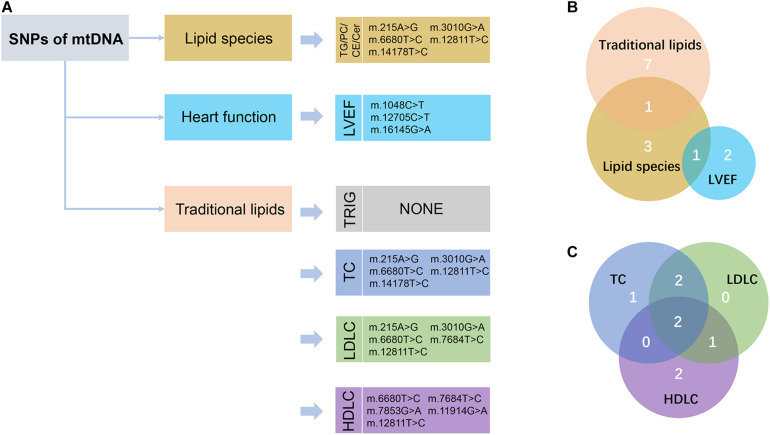
Schematic view of the association analyses results. **(A)** The nominally significant (*P* < 0.05) SNPs of mtDNA with lipid species, traditional lipids, and heart function were presented. TG, triacylglycerol; PC, phosphatidylcholines; CE, cholesteryl esters; Cer, ceramides; LVEF, left ventricular ejection fraction; TRIG, triglyceride; TC, total cholesterol; LDLC, low-density lipoprotein cholesterol; HDLC, high-density lipoprotein cholesterol. **(B)** Venn diagram showed the intersection of these nominally significant SNPs in lipid species, traditional lipids, and LVEF. **(C)** Venn diagram showed the intersection of the nominally significant SNPs in the traditional lipids, including TC, LDLC, and HDLC.

**FIGURE 4:**
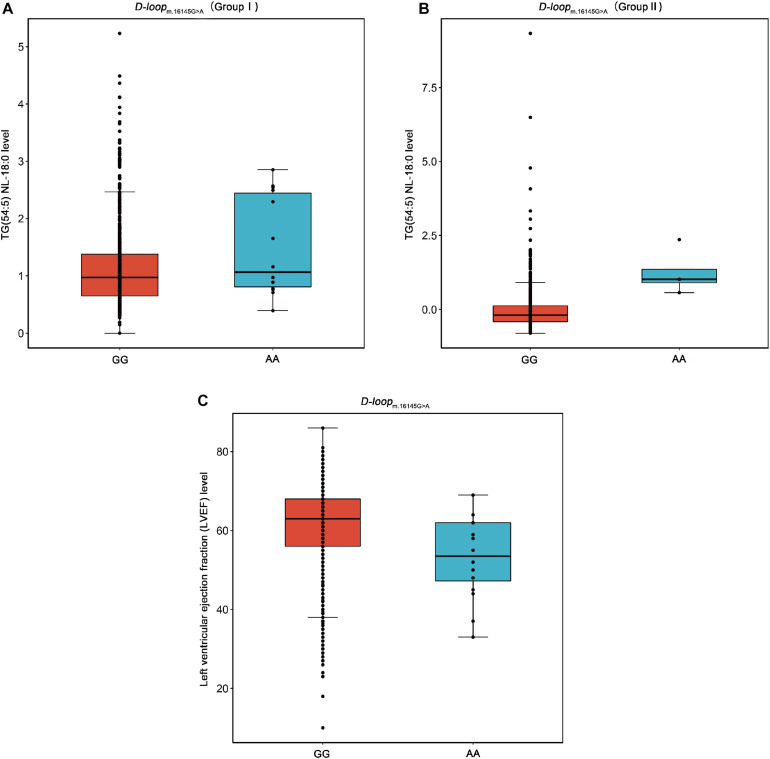
Box plots for the SNP m.16145G > A against the levels of TG(54:5) NL-18:0 and LVEF. TG, triacylglycerol; LVEF, left ventricular ejection fraction. **(A)** The SNP m.16145G > A (G > A) in D-loop against the level of TG(54:5) NL-18:0 in the Group I. **(B)** The SNP m.16145G > A (G > A) in D-loop against the level of TG(54:5) NL-18:0 in the Group II. **(C)** The SNP m.16145G > A (G > A) in D-loop against the level of LVEF.

We performed large-scale association analyses between mitochondrial genetic variants and lipidomic traits in a Chinese cohort with CAD. We identified two SNPs (m.16089T > C and m.16145G > A) of mtDNA that were statistically significantly associated with three lipid species (FDR < 0.05) in two independent groups. Then, we performed linear regression to identify the relationships of the SNPs of mtDNA with traditional lipids, and heart function to explore the influence of mitochondrial genetic variants with clinical manifestations. We found that two SNPs (m.14178T > C and m.215A > G) were statistically significantly associated with TC and LDLC (FDR < 0.05), and three SNPs (m.1048C > T, m.12705C > T, and m.16145G > A) were nominally significantly associated with LVEF (*P* < 0.05; Figure 3). The results may provide a potential theoretical basis for further explaining the large difference in the levels of lipids and poor prognosis in patients with CAD and provide potential drug intervention targets for precision medicine in patients with CAD.

m.16145G > A (G > A) associated with the increased level of TG(54:5) NL-18:0 but with the decreased level of LVEF were found in this study (Figure 4). This result suggests that the variation at m.16145G > A may bring adverse effect to patients with CAD. Interestingly, the SNPs of mtDNA that are statistically significantly related to the traditional lipids (FDR < 0.05) showed no significant correlation with LVEF (*P* > 0.05). The variation at the m.16145G > A was not statistically significantly related to the traditional lipids, suggesting the importance of studying mitochondrial genetic variants related to specific lipid species.

The SNPs of mtDNA were significantly associated with the level of lipid species (FDR < 0.05) in the D-loop. The D-loop region is a non-coding region but accumulates more mutations under increased oxidative stress than the other regions of mtDNA (Clayton, 2000). The initiation site for heavy chain replication and the promoter for heavy and light chain transcription are contained in this region (Chen et al., 2012). Therefore, the SNPs in the region of the D-loop may contain essential transcription and replication elements (Sharma et al., 2005). For example, the SNP m.16189T > C in D-loop is associated with vascular pathologies such as stroke and CAD (Liou et al., 2004; Mueller et al., 2011). We speculate that the variants in the D-loop region may affect the metabolism of lipid species, thereby affecting the cardiovascular prognosis.

By analyzing the correlation between mitochondrial genetic variation and plasma TC concentration, we found that m.14178T > C (T > C) in *MT-ND6* could significantly increase TC level (FDR < 0.001). The variation at m.14178T > C is a missense mutation that can change the encoded amino acid (T > C, isoleucine > valine), and *MT-ND6* is a mitochondrial gene that codes the NADH-ubiquinone oxidoreductase chain 6 protein (Shao et al., 2008). NADH is a reduced form of nicotinamide adenine dinucleotide, which is a cofactor in the metabolic center. NADH plays an important role in energy-producing functions of the mitochondria (Stein and Imai, 2012). Therefore, variations in *MT-ND6* may cause energy imbalance and metabolic diseases. A recent study reported that variants in *MT-ND6* can significantly increase the severity of cardiomyopathy (McManus et al., 2019). Our study found that the T to C base in *MT-ND6* m.14178T > C was associated with the increased level of plasma TC, thus possibly contributing to the occurrence and development of atherosclerosis. However, the causal relationship between this mutation and atherosclerosis is still unclear, and further research is needed.

Furthermore, we identified three SNPs that were nominally significantly associated with the level of LVEF (*P* < 0.05 but FDR > 0.05). The m.1048C > T in *MT-RNR1* was the most significant SNP of the results. *MT-RNR1* is an RNA gene that encodes 12S rRNA, which is mainly involved in the regulation of insulin sensitivity and metabolic balance (Lee et al., 2015). Previous studies reported that the SNP m.1555A > G in *MT-RNR1* was associated with atherosclerosis (Sobenin et al., 2012, 2013). We found that the mutation in this gene could affect heart function, suggesting that *MT-RNR1* may play an important role in the development of CAD. However, its relationship with LVEF level needs further analysis, and a large sample size is needed for the validation of this role.

Our present study has several limitations. Although we enrolled approximately 1,400 participants from three centers, a larger central cohort is needed for fully understanding the relationships between mitochondrial genetic variants and lipidomic profiling. Besides, the patients enrolled in this study were almost treated with statins for lipid-lowering medications, which may influence the plasma levels of cholesterol-derived lipids. Moreover, mitochondrial genetic data were obtained using GSA bead chips, and the number of SNPs detected was relatively small in this study. Increasing the number of SNPs and performing full mitochondrial sequencing could solve this problem in the future. Furthermore, our study can only provide the correlation, and the causality may need to be combined with causality analysis or other approaches, such as Mendelian randomization or molecular biology verification in the future.

## Conclusion

We performed large-scale association analyses in a Han Chinese population with CAD to explore the relationships between mitochondrial genetic variants and lipidomic profiling. We identified three statistically significant associations (FDR < 0.05), namely, m.16089T > C with TG(50:4) NL-16:0, m.16145G > A with TG(54:5) NL-18:0, and m.16089T > C with PC(16:0_16:1). Then, we further investigated the relationships between mitochondrial genetic variants and traditional lipids. Two statistically significant associations were found (FDR < 0.05), namely, m.14178T > C with TC and m.215A > G with LDLC. Furthermore, we performed association analyses on the SNPs of mtDNA and LVEF. We found that the association between the SNP m.16145G > A and LVEF was nominally significant (*P* = 0.047). These findings highlight the importance of studying mitochondrial genetic variants related to lipid species and could provide a potential target for pharmacological intervention and optimized treatment of patients with CAD.

## Data Availability Statement

The datasets presented in this study can be found in online repositories. The names of the repository/repositories and accession number(s) can be found below: EMBL-EBI (Project: PRJEB42554; Analyses: ERZ1714343).

## Ethics Statement

The studies involving human participants were reviewed and approved by the Medical Ethical Review Committee of Guangdong Provincial People’s Hospital (Nos. GDREC2010137 and GDREC2017071H) and conducted according to the Declaration of Helsinki. Informed consent (Nos. 20100910 and 20170211) was obtained from all individual participants included in the study. The patients/participants provided their written informed consent to participate in this study.

## Author Contributions

ZW designed the study, performed the data analysis, and wrote the manuscript. HC participated in patient recruitment, performed the experiment, and revised the manuscript. MQ performed the experiment and the data analysis. CL, QM, and XC curated the data. YZ made the investigation. WL provided the resources. XZ and SZ designed the study, leaded the study, and provided the resources. All authors reviewed and approved the final manuscript.

## Conflict of Interest

The authors declare that the research was conducted in the absence of any commercial or financial relationships that could be construed as a potential conflict of interest.

## Footnotes

1 https://www.r-project.org/

## Abbreviations

CAD: coronary artery disease
SNP: single nucleotide polymorphism
mtDNA: mitochondrial DNA
TRIG: triglyceride
TC: total cholesterol
LDLC: low-density lipoprotein cholesterol
HDLC: high-density lipoprotein cholesterol
LVEF: left ventricular ejection fraction
CVD: cardiovascular disease
D-loop: displacement loop
PCI: percutaneous coronary intervention
MG: monoglyceride
CE: cholesteryl esters
DG: diacylglycerol
TG: triacylglycerol
PA: phosphatidic acids
PC: phosphatidylcholines
PG: phosphatidylglycerol
PS: phosphatidylserines
PE: phosphatidylethanolamines
LPA: lysophosphatidic acids
LPC: lysophosphatidylcholine
LPE: lysophosphatidylethanolamine
LPS: hemolytic serine
Cer: ceramides
GSA: global screening array
AST: aspartate aminotransferase
eGFR: estimated glomerular filtration rate
FDR: false discovery rate.
