# Supplementary material for: Associations of Mitochondrial Variants With Lipidomic Traits in a Chinese Cohort With Coronary Artery Disease
Source: Front Genet. 2021 Mar 25;12:630359. doi: 10.3389/fgene.2021.630359 (PMC8027325; doi:10.3389/fgene.2021.630359)
Supplement: Supplementary file 1 [file Data_Sheet_1.docx]

**Supplementary Materials**

**Associations of** **mitochondrial variants with lipidomic traits in a Chinese cohort with coronary artery disease**

**Running title:** Associations between mtDNA and lipidomic

Zixian Wang ^1, 2, 3, 4 #^, Hui Chen ^1, 2, 3 #^, Min Qin ^1, 2, 3^, Chen Liu ^5^, Qilin Ma ^6^, Xiaoping Chen ^7^, Ying Zhang ^8^, Weihua Lai ^2^, Xiaojuan Zhang ^2 *^, Shilong Zhong ^1, 2, 3, 4 *^

# The authors contributed equally to the study and are considered co-first authors.

* Correspondence author:

Shilong Zhong

Department of Pharmacy,

Guangdong Provincial Key Laboratory of Coronary Heart Disease Prevention,

Guangdong Provincial People's Hospital,

Guangdong Academy of Medical Sciences.

106 Zhongshan Road,

Guangzhou 510080, P. R. China

Tel: +8620-83827812 - 60298

Email: [gdph_zhongsl@gd.gov.cn](mailto:gdph_zhongsl@gd.gov.cn)

and

Xiaojuan Zhang

Department of Pharmacy,

Guangdong Provincial People's Hospital,

Guangdong Academy of Medical Sciences.

106 Zhongshan Road,

Guangzhou 510080, P. R. China

Tel: +8620-83827812 – 36066

Email: [zhangxjtj@163.com](mailto:zhangxjtj@163.com)

**Widely targeted lipidomic profiling**

In the Group Ⅰ and Group Ⅱ sets, the widely targeted lipidomic profiling was performed using ultra-performance liquid chromatography mass spectrometry (UPLC-MS/MS) system (UPLC, Shim-pack UFLC SHIMADZU CBM30A; MS, Applied Biosystems SCIEX 6500+ QTRAP) at Wuhan Metware Biotechnology. Totally, 667 plasma endogenous lipid species consisting of 14 lipid classes/subclasses and 687 lipid species containing 20 lipid classes/subclasses were annotated in the Group Ⅰ and Group Ⅱ, respectively. Eventually, 309 identical lipid species were detected in both groups. The ESI full scan mass spectra ion pairs and conditions for tandem mass spectrometry analysis of the lipid species are shown in Table S6.

Lipid species were extracted from the plasma of CAD patients. Firstly, the sample was thawed on ice, whirled for 10 s and centrifuged with 3000 r/min at 4 ℃ for 5 min. Secondly, 50 μL of plasma and 1 mL of lipid extraction reagent were pooled into the corresponding numbered centrifuge tube. The mixture was vortexed for 2 min, added with 500 μL of deionised water, vortexed for 1 min and centrifuged with 12,000 r/min at 4 ℃ for 10 min. Thirdly, 500 μL of supernatant was absorbed into the numbered centrifuge tube and concentrated after centrifugation. Lastly, the powder was dissolved with 100 μL of mobile phase B (comprising 10% acetonitrile, 90% isopropanol, 0.04% acetic acid, and 5 mmol/L ammonium formate), and the dissolving solution was then used for UPLC-MS/MS analysis.

The calibration and quality control (QC) samples were prepared with the mixed plasma of subjects prior to sample analysis. Every 10 samples to be analysed were separated by one QC sample for the duration of the detection to monitor repeatability during the analysis. The repeatability of lipid extraction and detection was judged by the overlapping analysis of total ion flow diagrams between different QC samples. The high overlaps of the total ion flow, that is, the retention time and peak strength are consistent, indicates that the signal stability of the mass spectrum is good at different times.

The separation was performed in a Thermo C30 column (2.6 µM, 2.1 mm × 100 mm). A UPLC column (Thermo C30, 2.6 μm, 2.1 mm*100 mm) was used at a column temperature of 45〬C. The mobile phase was composed of acetonitrile/water (60/40, V/V) containing 0.04% acetic acid, 5 mmol/L ammonium formate (A) and acetonitrile/isopropanol (10/90, V/V) containing 0.04% acetic acid and 5 mmol/L ammonium formate (B). The gradient program initiated from 20% B to 50% B at 3.0 min, to 65% B at 5 min, to 75% B at 9 min and to 90% B at 15.5 min with a flow rate at 0.35 mL/min. The injection volume was set at 2 μL. The effluent was alternatively connected to an ESI-QTRAP-MS.

The LIT and triple quadrupole (QQQ) scans were acquired on a QTRAP-MS (QTRAP® 6500+ LC-MS/MS System) equipped with an ESI Turbo Ion-Spray interface operating in positive and negative ion modes and controlled by Analyst 1.6.3 software (Sciex). The ESI source operation parameters were as follows: ion source, turbo spray; source temperature of 550 °C and ion spray voltage of 5500 V. The ion source gas I, gas II and curtain gas were set at 55, 60, and 25 psi, respectively. The collision gas was medium. Instrument tuning and mass calibration were performed with 10 and 100 μmol/L polypropylene glycol solutions in QQQ and LIT modes, respectively. The QQQ scans were acquired as MRM experiments with collision gas (nitrogen) set to 5 psi. The DP and CE for individual MRM transitions were conducted with further DP and CE optimisation. A specific set of MRM transitions were monitored for each period according to the metabolites eluted within this period.

Qualitative analysis of the MS and MS/MS mass spectrometric data was performed on the basis of the home-made database Metware database (MWDB) and the public database of metabolite information. The lipid metabolite structural analysis mainly referred to MassBank, HMDB, LIPID MAPS and METLIN database. Analyst 1.6.3 software (AB Sciex) was used to process the raw mass spectrometry data.

**Supplementary Tables Legends**

Table S1. All association results of mtDNA variations with triglyceride.

Table S2. All association results of mtDNA variations with total cholesterol.

Table S3. All association results of mtDNA variations with low-density lipoprotein cholesterol.

Table S4. All association results of mtDNA variations with high-density lipoprotein cholesterol.

Table S5. All association results of mtDNA variations with left ventricular ejection fraction.

Table S6. Conditions for tandem mass spectrometry analysis of lipid species.

| **Table S1. All association results of mtDNA variations with triglyceride.** | | | | | | | | |
| --- | --- | --- | --- | --- | --- | --- | --- | --- |
| **Position** | **mtSNP** | **Gene/region** | **Test** | **N** | **Effect** | **SE** | **P** | **FDR** |
| 14979 | m.14979T>C | *CYTB* | T>C | 1388 | 0.38 | 0.22 | 0.086020 | 0.974297 |
| 11215 | m.11215C>T | *MT-ND4* | C>T | 1409 | -0.42 | 0.26 | 0.103750 | 0.974297 |
| 3206 | m.3206C>T | *MT-RNR2* | C>T | 1400 | 0.35 | 0.22 | 0.105384 | 0.974297 |
| 15487 | m.15487A>T | *CYTB* | A>T | 1409 | 0.19 | 0.15 | 0.210690 | 0.974297 |
| 1048 | m.1048C>T | *MT-RNR1* | C>T | 1403 | -0.32 | 0.28 | 0.249537 | 0.974297 |
| 14178 | m.14178T>C | *MT-ND6* | T>C | 1409 | -0.38 | 0.35 | 0.277188 | 0.974297 |
| 16129 | m.16129G>A | *D-loop* | G>A | 1392 | 0.09 | 0.09 | 0.286374 | 0.974297 |
| 13105 | m.13105A>G | *MT-ND5* | A>G | 1408 | -0.35 | 0.34 | 0.293150 | 0.974297 |
| 16145 | m.16145G>A | *D-loop* | G>A | 1409 | 0.35 | 0.34 | 0.298543 | 0.974297 |
| 10397 | m.10397A>G | *MT-ND3* | A>G | 1409 | -0.18 | 0.17 | 0.300556 | 0.974297 |
| 5108 | m.5108T>C | *MT-ND2* | T>C | 1409 | -0.23 | 0.22 | 0.301832 | 0.974297 |
| 12705 | m.12705C>T | *MT-ND5* | T>C | 1403 | 0.08 | 0.08 | 0.307481 | 0.974297 |
| 5301 | m.5301A>G | *MT-ND2* | A>G | 1406 | -0.18 | 0.18 | 0.320760 | 0.974297 |
| 15535 | m.15535C>T | *CYTB* | C>T | 1409 | -0.18 | 0.18 | 0.330769 | 0.974297 |
| 1438 | m.1438G>A | *MT-RNR1* | G>A | 1352 | -0.20 | 0.24 | 0.400146 | 0.974297 |
| 16162 | m.16162A>G | *D-loop* | A>G | 1409 | 0.11 | 0.16 | 0.477517 | 0.974297 |
| 7853 | m.7853G>A | *MT-CO2* | G>A | 1406 | -0.08 | 0.12 | 0.490557 | 0.974297 |
| 16217 | m.16217T>C | *D-loop* | T>C | 1367 | 0.07 | 0.12 | 0.527872 | 0.974297 |
| 3010 | m.3010G>A | *MT-RNR2* | G>A | 1407 | 0.07 | 0.12 | 0.573633 | 0.974297 |
| 12630 | m.12630G>A | *MT-ND5* | G>A | 1409 | -0.14 | 0.28 | 0.602426 | 0.974297 |
| 215 | m.215A>G | *D-loop* | A>G | 1399 | 0.18 | 0.37 | 0.620883 | 0.974297 |
| 12882 | m.12882C>T | *MT-ND5* | C>T | 1405 | -0.05 | 0.11 | 0.640767 | 0.974297 |
| 7684 | m.7684T>C | *MT-CO2* | T>C | 1409 | -0.06 | 0.12 | 0.642294 | 0.974297 |
| 523 | m.523A>C | *D-loop* | A>C | 1377 | -0.03 | 0.08 | 0.656423 | 0.974297 |
| 5442 | m.5442T>C | *MT-ND2* | T>C | 1400 | 0.07 | 0.19 | 0.713069 | 0.974297 |
| 6680 | m.6680T>C | *MT-CO1* | T>C | 1406 | -0.05 | 0.13 | 0.717594 | 0.974297 |
| 12811 | m.12811T>C | *MT-ND5* | T>C | 1370 | -0.05 | 0.13 | 0.719902 | 0.974297 |
| 12771 | m.12771G>A | *MT-ND5* | G>A | 1409 | -0.08 | 0.25 | 0.738959 | 0.974297 |
| 13263 | m.13263A>G | *MT-ND5* | A>G | 1408 | 0.07 | 0.23 | 0.765386 | 0.974297 |
| 11914 | m.11914G>A | *MT-ND4* | G>A | 1400 | 0.05 | 0.17 | 0.786335 | 0.974297 |
| 489 | m.489T>C | *D-loop* | T>C | 1406 | -0.02 | 0.08 | 0.790211 | 0.974297 |
| 8020 | m.8020G>A | *MT-CO2* | G>A | 1407 | -0.06 | 0.25 | 0.806107 | 0.974297 |
| 15670 | m.15670T>C | *CYTB* | T>C | 1407 | -0.07 | 0.31 | 0.811309 | 0.974297 |
| 15924 | m.15924A>G | *tRNA-Thr* | A>G | 1408 | -0.07 | 0.34 | 0.841800 | 0.974297 |
| 8964 | m.8964C>T | *MT-ATP6* | C>T | 1409 | -0.06 | 0.34 | 0.849114 | 0.974297 |
| 15043 | m.15043G>A | *CYTB* | G>A | 1406 | 0.01 | 0.08 | 0.903782 | 0.974297 |
| 16086 | m.16089T>C | *D-loop* | T>C | 1406 | 0.02 | 0.27 | 0.927663 | 0.974297 |
| 16327 | m.16327C>T | *D-loop* | C>T | 1407 | 0.02 | 0.22 | 0.937954 | 0.974297 |
| 6392 | m.6392T>C | *MT-CO1* | T>C | 1408 | 0.01 | 0.09 | 0.940909 | 0.974297 |
| 14502 | m.14502T>C | *MT-ND6* | T>C | 1408 | 0.02 | 0.27 | 0.950377 | 0.974297 |
| 11536 | m.11536C>T | *MT-ND4* | C>T | 1409 | 0.02 | 0.37 | 0.951099 | 0.974297 |
| 3394 | m.3394T>C | *MT-ND1* | T>C | 1402 | 0.00 | 0.25 | 0.993492 | 0.993492 |
| N = number of test; SE = standard error; FDR = false discovery rate. | | | | | | | | |

| **Table S2. All association results of mtDNA variations with total cholesterol.** | | | | | | | | |
| --- | --- | --- | --- | --- | --- | --- | --- | --- |
| **Position** | **mtSNP** | **Gene/region** | **Test** | **N** | **Effect** | **SE** | **P** | **FDR** |
| 14178 | m.14178T>C | *MT-ND6* | T>C | 1409 | 1.45 | 0.34 | 0.000016 | 0.000691 |
| 215 | m.215A>G | *D-loop* | A>G | 1399 | 1.08 | 0.36 | 0.002684 | 0.056354 |
| 6680 | m.6680T>C | *MT-CO1* | T>C | 1406 | 0.28 | 0.13 | 0.026426 | 0.369958 |
| 12811 | m.12811T>C | *MT-ND5* | T>C | 1370 | 0.27 | 0.13 | 0.036252 | 0.380642 |
| 3010 | m.3010G>A | *MT-RNR2* | G>A | 1407 | -0.23 | 0.12 | 0.045509 | 0.382271 |
| 7684 | m.7684T>C | *MT-CO2* | T>C | 1409 | 0.21 | 0.12 | 0.072201 | 0.505410 |
| 8964 | m.8964C>T | *MT-ATP6* | C>T | 1409 | -0.56 | 0.33 | 0.088598 | 0.528901 |
| 14502 | m.14502T>C | *MT-ND6* | T>C | 1408 | -0.43 | 0.26 | 0.100743 | 0.528901 |
| 11914 | m.11914G>A | *MT-ND4* | G>A | 1400 | -0.23 | 0.16 | 0.168701 | 0.705971 |
| 16162 | m.16162A>G | *D-loop* | A>G | 1409 | -0.22 | 0.16 | 0.169410 | 0.705971 |
| 12771 | m.12771G>A | *MT-ND5* | G>A | 1409 | -0.32 | 0.24 | 0.186758 | 0.705971 |
| 7853 | m.7853G>A | *MT-CO2* | G>A | 1406 | 0.14 | 0.11 | 0.217230 | 0.705971 |
| 13263 | m.13263A>G | *MT-ND5* | A>G | 1408 | -0.26 | 0.22 | 0.235016 | 0.705971 |
| 11215 | m.11215C>T | *MT-ND4* | C>T | 1409 | -0.29 | 0.25 | 0.251223 | 0.705971 |
| 16327 | m.16327C>T | *D-loop* | C>T | 1407 | -0.24 | 0.21 | 0.254010 | 0.705971 |
| 523 | m.523A>C | *D-loop* | A>C | 1377 | -0.08 | 0.08 | 0.269956 | 0.705971 |
| 12882 | m.12882C>T | *MT-ND5* | C>T | 1405 | -0.12 | 0.11 | 0.285750 | 0.705971 |
| 3394 | m.3394T>C | *MT-ND1* | T>C | 1402 | -0.24 | 0.24 | 0.315219 | 0.716858 |
| 8020 | m.8020G>A | *MT-CO2* | G>A | 1407 | -0.24 | 0.24 | 0.324293 | 0.716858 |
| 1048 | m.1048C>T | *MT-RNR1* | C>T | 1403 | 0.21 | 0.27 | 0.443060 | 0.885749 |
| 11536 | m.11536C>T | *MT-ND4* | C>T | 1409 | 0.25 | 0.36 | 0.491463 | 0.885749 |
| 3206 | m.3206C>T | *MT-RNR2* | C>T | 1400 | -0.14 | 0.21 | 0.507707 | 0.885749 |
| 14979 | m.14979T>C | *CYTB* | T>C | 1388 | -0.14 | 0.22 | 0.513466 | 0.885749 |
| 12705 | m.12705C>T | *MT-ND5* | T>C | 1403 | -0.05 | 0.07 | 0.539860 | 0.885749 |
| 5108 | m.5108T>C | *MT-ND2* | T>C | 1409 | -0.13 | 0.22 | 0.559860 | 0.885749 |
| 15670 | m.15670T>C | *CYTB* | T>C | 1407 | 0.17 | 0.30 | 0.574047 | 0.885749 |
| 5301 | m.5301A>G | *MT-ND2* | A>G | 1406 | 0.09 | 0.17 | 0.588975 | 0.885749 |
| 15487 | m.15487A>T | *CYTB* | A>T | 1409 | -0.08 | 0.15 | 0.590499 | 0.885749 |
| 16086 | m.16089T>C | *D-loop* | T>C | 1406 | 0.13 | 0.26 | 0.620359 | 0.898451 |
| 15924 | m.15924A>G | *tRNA-Thr* | A>G | 1408 | -0.15 | 0.33 | 0.652094 | 0.910972 |
| 6392 | m.6392T>C | *MT-CO1* | T>C | 1408 | -0.04 | 0.09 | 0.676381 | 0.910972 |
| 10397 | m.10397A>G | *MT-ND3* | A>G | 1409 | 0.07 | 0.17 | 0.696459 | 0.910972 |
| 1438 | m.1438G>A | *MT-RNR1* | G>A | 1352 | -0.08 | 0.24 | 0.744735 | 0.910972 |
| 13105 | m.13105A>G | *MT-ND5* | A>G | 1408 | -0.10 | 0.33 | 0.758117 | 0.910972 |
| 5442 | m.5442T>C | *MT-ND2* | T>C | 1400 | 0.06 | 0.19 | 0.759143 | 0.910972 |
| 16217 | m.16217T>C | *D-loop* | T>C | 1367 | -0.02 | 0.11 | 0.824336 | 0.937336 |
| 489 | m.489T>C | *D-loop* | T>C | 1406 | 0.02 | 0.07 | 0.825748 | 0.937336 |
| 16129 | m.16129G>A | *D-loop* | G>A | 1392 | -0.01 | 0.08 | 0.864177 | 0.955143 |
| 15043 | m.15043G>A | *CYTB* | G>A | 1406 | 0.01 | 0.07 | 0.926508 | 0.990638 |
| 16145 | m.16145G>A | *D-loop* | G>A | 1409 | -0.02 | 0.33 | 0.961848 | 0.990638 |
| 12630 | m.12630G>A | *MT-ND5* | G>A | 1409 | 0.00 | 0.27 | 0.986494 | 0.990638 |
| 15535 | m.15535C>T | *CYTB* | C>T | 1409 | 0.00 | 0.18 | 0.990638 | 0.990638 |
| N = number of tests; SE = standard error; FDR = false discovery rate. | | | | | | | | |

| **Table S3. All association results of mtDNA variations with low-density lipoprotein cholesterol.** | | | | | | | | |
| --- | --- | --- | --- | --- | --- | --- | --- | --- |
| **Position** | **mtSNP** | **Gene/region** | **Test** | **N** | **Effect** | **SE** | **P** | **FDR** |
| 215 | m.215A>G | *D-loop* | A>G | 1399 | 0.80 | 0.24 | 0.001098 | 0.046129 |
| 6680 | m.6680T>C | *MT-CO1* | T>C | 1406 | 0.25 | 0.09 | 0.002930 | 0.061529 |
| 12811 | m.12811T>C | *MT-ND5* | T>C | 1370 | 0.24 | 0.09 | 0.006208 | 0.086907 |
| 3010 | m.3010G>A | *MT-RNR2* | G>A | 1407 | -0.20 | 0.08 | 0.012639 | 0.132706 |
| 7684 | m.7684T>C | *MT-CO2* | T>C | 1409 | 0.19 | 0.08 | 0.015906 | 0.133611 |
| 16162 | m.16162A>G | *D-loop* | A>G | 1409 | -0.20 | 0.11 | 0.057785 | 0.344573 |
| 7853 | m.7853G>A | *MT-CO2* | G>A | 1406 | 0.14 | 0.08 | 0.066785 | 0.344573 |
| 5301 | m.5301A>G | *MT-ND2* | A>G | 1406 | 0.21 | 0.12 | 0.071569 | 0.344573 |
| 489 | m.489T>C | *D-loop* | T>C | 1406 | 0.09 | 0.05 | 0.073837 | 0.344573 |
| 1048 | m.1048C>T | *MT-RNR1* | C>T | 1403 | 0.31 | 0.19 | 0.096150 | 0.401919 |
| 10397 | m.10397A>G | *MT-ND3* | A>G | 1409 | 0.18 | 0.11 | 0.120420 | 0.401919 |
| 523 | m.523A>C | *D-loop* | A>C | 1377 | -0.08 | 0.05 | 0.123422 | 0.401919 |
| 8964 | m.8964C>T | *MT-ATP6* | C>T | 1409 | -0.34 | 0.22 | 0.130714 | 0.401919 |
| 15043 | m.15043G>A | *CYTB* | G>A | 1406 | 0.08 | 0.05 | 0.133973 | 0.401919 |
| 14502 | m.14502T>C | *MT-ND6* | T>C | 1408 | -0.26 | 0.18 | 0.156900 | 0.439320 |
| 12882 | m.12882C>T | *MT-ND5* | C>T | 1405 | -0.09 | 0.07 | 0.213981 | 0.526189 |
| 12705 | m.12705C>T | *MT-ND5* | T>C | 1403 | -0.06 | 0.05 | 0.223624 | 0.526189 |
| 11215 | m.11215C>T | *MT-ND4* | C>T | 1409 | -0.21 | 0.17 | 0.225867 | 0.526189 |
| 12771 | m.12771G>A | *MT-ND5* | G>A | 1409 | -0.19 | 0.16 | 0.238038 | 0.526189 |
| 16086 | m.16089T>C | *D-loop* | T>C | 1406 | 0.19 | 0.18 | 0.279678 | 0.578822 |
| 11914 | m.11914G>A | *MT-ND4* | G>A | 1400 | -0.12 | 0.11 | 0.295781 | 0.578822 |
| 13263 | m.13263A>G | *MT-ND5* | A>G | 1408 | -0.15 | 0.15 | 0.308935 | 0.578822 |
| 14979 | m.14979T>C | *CYTB* | T>C | 1388 | -0.14 | 0.15 | 0.346020 | 0.578822 |
| 16327 | m.16327C>T | *D-loop* | C>T | 1407 | -0.13 | 0.14 | 0.366761 | 0.578822 |
| 15535 | m.15535C>T | *CYTB* | C>T | 1409 | 0.10 | 0.12 | 0.400947 | 0.578822 |
| 8020 | m.8020G>A | *MT-CO2* | G>A | 1407 | -0.14 | 0.16 | 0.401278 | 0.578822 |
| 14178 | m.14178T>C | *MT-ND6* | T>C | 1409 | -0.19 | 0.23 | 0.405177 | 0.578822 |
| 3206 | m.3206C>T | *MT-RNR2* | C>T | 1400 | -0.12 | 0.15 | 0.409817 | 0.578822 |
| 15670 | m.15670T>C | *CYTB* | T>C | 1407 | 0.17 | 0.21 | 0.423083 | 0.578822 |
| 1438 | m.1438G>A | *MT-RNR1* | G>A | 1352 | 0.12 | 0.15 | 0.424962 | 0.578822 |
| 5442 | m.5442T>C | *MT-ND2* | T>C | 1400 | 0.10 | 0.13 | 0.427226 | 0.578822 |
| 15924 | m.15924A>G | *tRNA-Thr* | A>G | 1408 | -0.16 | 0.22 | 0.462982 | 0.607664 |
| 11536 | m.11536C>T | *MT-ND4* | C>T | 1409 | 0.16 | 0.24 | 0.501006 | 0.636772 |
| 16145 | m.16145G>A | *D-loop* | G>A | 1409 | -0.15 | 0.22 | 0.515482 | 0.636772 |
| 13105 | m.13105A>G | *MT-ND5* | A>G | 1408 | -0.13 | 0.22 | 0.556149 | 0.666555 |
| 5108 | m.5108T>C | *MT-ND2* | T>C | 1409 | -0.08 | 0.15 | 0.571333 | 0.666555 |
| 12630 | m.12630G>A | *MT-ND5* | G>A | 1409 | 0.09 | 0.18 | 0.612130 | 0.677954 |
| 3394 | m.3394T>C | *MT-ND1* | T>C | 1402 | -0.08 | 0.16 | 0.613387 | 0.677954 |
| 6392 | m.6392T>C | *MT-CO1* | T>C | 1408 | -0.03 | 0.06 | 0.632465 | 0.681116 |
| 16217 | m.16217T>C | *D-loop* | T>C | 1367 | -0.03 | 0.08 | 0.654750 | 0.687488 |
| 16129 | m.16129G>A | *D-loop* | G>A | 1392 | 0.02 | 0.06 | 0.747160 | 0.765383 |
| 15487 | m.15487A>T | *CYTB* | A>T | 1409 | -0.01 | 0.10 | 0.884781 | 0.884781 |
| N = number of tests; SE = standard error; FDR = false discovery rate. | | | | | | | | |

| **Table S4. All association results of mtDNA variations with high-density lipoprotein cholesterol.** | | | | | | | | |
| --- | --- | --- | --- | --- | --- | --- | --- | --- |
| **Position** | **mtSNP** | **Gene/region** | **Test** | **N** | **Effect** | **SE** | **P** | **FDR** |
| 6680 | m.6680T>C | *MT-CO1* | T>C | 1406 | 0.06 | 0.02 | 0.009879 | 0.203592 |
| 12811 | m.12811T>C | *MT-ND5* | T>C | 1370 | 0.06 | 0.02 | 0.010075 | 0.203592 |
| 7684 | m.7684T>C | *MT-CO2* | T>C | 1409 | 0.05 | 0.02 | 0.014542 | 0.203592 |
| 7853 | m.7853G>A | *MT-CO2* | G>A | 1406 | 0.05 | 0.02 | 0.025186 | 0.245955 |
| 11914 | m.11914G>A | *MT-ND4* | G>A | 1400 | -0.06 | 0.03 | 0.029280 | 0.245955 |
| 1438 | m.1438G>A | *MT-RNR1* | G>A | 1352 | -0.08 | 0.04 | 0.063765 | 0.379883 |
| 14502 | m.14502T>C | *MT-ND6* | T>C | 1408 | -0.08 | 0.05 | 0.079134 | 0.379883 |
| 11536 | m.11536C>T | *MT-ND4* | C>T | 1409 | 0.11 | 0.06 | 0.079526 | 0.379883 |
| 14178 | m.14178T>C | *MT-ND6* | T>C | 1409 | 0.10 | 0.06 | 0.081404 | 0.379883 |
| 5301 | m.5301A>G | *MT-ND2* | A>G | 1406 | -0.05 | 0.03 | 0.127877 | 0.466806 |
| 3206 | m.3206C>T | *MT-RNR2* | C>T | 1400 | -0.06 | 0.04 | 0.139538 | 0.466806 |
| 15535 | m.15535C>T | *CYTB* | C>T | 1409 | -0.05 | 0.03 | 0.143721 | 0.466806 |
| 10397 | m.10397A>G | *MT-ND3* | A>G | 1409 | -0.04 | 0.03 | 0.148716 | 0.466806 |
| 13263 | m.13263A>G | *MT-ND5* | A>G | 1408 | -0.06 | 0.04 | 0.155602 | 0.466806 |
| 14979 | m.14979T>C | *CYTB* | T>C | 1388 | -0.05 | 0.04 | 0.175403 | 0.491128 |
| 215 | m.215A>G | *D-loop* | A>G | 1399 | 0.07 | 0.06 | 0.236913 | 0.591096 |
| 16327 | m.16327C>T | *D-loop* | C>T | 1407 | -0.04 | 0.04 | 0.239253 | 0.591096 |
| 3394 | m.3394T>C | *MT-ND1* | T>C | 1402 | -0.05 | 0.04 | 0.272262 | 0.635278 |
| 8964 | m.8964C>T | *MT-ATP6* | C>T | 1409 | -0.06 | 0.06 | 0.308429 | 0.655422 |
| 12771 | m.12771G>A | *MT-ND5* | G>A | 1409 | -0.04 | 0.04 | 0.323151 | 0.655422 |
| 12882 | m.12882C>T | *MT-ND5* | C>T | 1405 | -0.02 | 0.02 | 0.327711 | 0.655422 |
| 3010 | m.3010G>A | *MT-RNR2* | G>A | 1407 | -0.02 | 0.02 | 0.347828 | 0.664035 |
| 16217 | m.16217T>C | *D-loop* | T>C | 1367 | -0.02 | 0.02 | 0.417024 | 0.745253 |
| 12705 | m.12705C>T | *MT-ND5* | T>C | 1403 | -0.01 | 0.01 | 0.425859 | 0.745253 |
| 1048 | m.1048C>T | *MT-RNR1* | C>T | 1403 | -0.03 | 0.05 | 0.472784 | 0.794277 |
| 15924 | m.15924A>G | *tRNA-Thr* | A>G | 1408 | 0.04 | 0.06 | 0.509279 | 0.822681 |
| 16129 | m.16129G>A | *D-loop* | G>A | 1392 | -0.01 | 0.01 | 0.531486 | 0.826756 |
| 15487 | m.15487A>T | *CYTB* | A>T | 1409 | -0.01 | 0.03 | 0.602365 | 0.903548 |
| 489 | m.489T>C | *D-loop* | T>C | 1406 | 0.01 | 0.01 | 0.640878 | 0.928168 |
| 8020 | m.8020G>A | *MT-CO2* | G>A | 1407 | -0.02 | 0.04 | 0.674048 | 0.938893 |
| 12630 | m.12630G>A | *MT-ND5* | G>A | 1409 | -0.02 | 0.05 | 0.725260 | 0.938893 |
| 13105 | m.13105A>G | *MT-ND5* | A>G | 1408 | 0.02 | 0.06 | 0.739526 | 0.938893 |
| 15043 | m.15043G>A | *CYTB* | G>A | 1406 | 0.00 | 0.01 | 0.768852 | 0.938893 |
| 16162 | m.16162A>G | *D-loop* | A>G | 1409 | -0.01 | 0.03 | 0.774106 | 0.938893 |
| 523 | m.523A>C | *D-loop* | A>C | 1377 | 0.00 | 0.01 | 0.782411 | 0.938893 |
| 6392 | m.6392T>C | *MT-CO1* | T>C | 1408 | 0.00 | 0.02 | 0.808720 | 0.943507 |
| 16145 | m.16145G>A | *D-loop* | G>A | 1409 | 0.01 | 0.06 | 0.863844 | 0.980580 |
| 16086 | m.16089T>C | *D-loop* | T>C | 1406 | 0.00 | 0.05 | 0.966738 | 0.996522 |
| 5108 | m.5108T>C | *MT-ND2* | T>C | 1409 | 0.00 | 0.04 | 0.971232 | 0.996522 |
| 11215 | m.11215C>T | *MT-ND4* | C>T | 1409 | 0.00 | 0.04 | 0.974065 | 0.996522 |
| 5442 | m.5442T>C | *MT-ND2* | T>C | 1400 | 0.00 | 0.03 | 0.995714 | 0.996522 |
| 15670 | m.15670T>C | *CYTB* | T>C | 1407 | 0.00 | 0.05 | 0.996522 | 0.996522 |
| N = number of tests; SE = standard error; FDR = false discovery rate. | | | | | | | | |

| **Table S5. All association results of mtDNA variations with left ventricular ejection fraction.** | | | | | | | | |
| --- | --- | --- | --- | --- | --- | --- | --- | --- |
| **Position** | **mtSNP** | **Gene/region** | **Test** | **N** | **Effect** | **SE** | **P** | **FDR** |
| 1048 | m.1048C>T | *MT-RNR1* | C>T | 1146 | -5.84 | 2.40 | 0.015093 | 0.633898 |
| 12705 | m.12705C>T | *MT-ND5* | T>C | 1145 | -1.37 | 0.66 | 0.038990 | 0.658633 |
| 16145 | m.16145G>A | *D-loop* | G>A | 1151 | -5.58 | 2.81 | 0.047045 | 0.658633 |
| 15043 | m.15043G>A | *CYTB* | G>A | 1149 | 1.11 | 0.66 | 0.095717 | 0.775928 |
| 489 | m.489T>C | *D-loop* | T>C | 1149 | 1.05 | 0.66 | 0.115665 | 0.775928 |
| 3394 | m.3394T>C | *MT-ND1* | T>C | 1144 | 3.47 | 2.31 | 0.132831 | 0.775928 |
| 14502 | m.14502T>C | *MT-ND6* | T>C | 1150 | 3.42 | 2.41 | 0.156295 | 0.775928 |
| 5301 | m.5301A>G | *MT-ND2* | A>G | 1148 | -2.12 | 1.54 | 0.170674 | 0.775928 |
| 11536 | m.11536C>T | *MT-ND4* | C>T | 1151 | -4.32 | 3.39 | 0.203412 | 0.775928 |
| 15535 | m.15535C>T | *CYTB* | C>T | 1151 | -2.09 | 1.70 | 0.221204 | 0.775928 |
| 15670 | m.15670T>C | *CYTB* | T>C | 1149 | 3.17 | 2.73 | 0.245482 | 0.775928 |
| 6392 | m.6392T>C | *MT-CO1* | T>C | 1150 | -0.95 | 0.83 | 0.249057 | 0.775928 |
| 10397 | m.10397A>G | *MT-ND3* | A>G | 1151 | -1.66 | 1.47 | 0.259057 | 0.775928 |
| 16162 | m.16162A>G | *D-loop* | A>G | 1151 | -1.61 | 1.43 | 0.260265 | 0.775928 |
| 16217 | m.16217T>C | *D-loop* | T>C | 1116 | -1.01 | 1.00 | 0.312408 | 0.775928 |
| 12882 | m.12882C>T | *MT-ND5* | C>T | 1147 | -0.98 | 0.98 | 0.319171 | 0.775928 |
| 16129 | m.16129G>A | *D-loop* | G>A | 1137 | 0.72 | 0.74 | 0.330974 | 0.775928 |
| 15924 | m.15924A>G | *tRNA-Thr* | A>G | 1150 | -2.75 | 3.01 | 0.361098 | 0.775928 |
| 12771 | m.12771G>A | *MT-ND5* | G>A | 1151 | 1.98 | 2.22 | 0.370868 | 0.775928 |
| 13105 | m.13105A>G | *MT-ND5* | A>G | 1150 | -2.55 | 2.92 | 0.381514 | 0.775928 |
| 15487 | m.15487A>T | *CYTB* | A>T | 1151 | 1.11 | 1.28 | 0.387964 | 0.775928 |
| 523 | m.523A>C | *D-loop* | A>C | 1123 | -0.50 | 0.67 | 0.458639 | 0.873973 |
| 5442 | m.5442T>C | *MT-ND2* | T>C | 1144 | 1.24 | 1.75 | 0.478604 | 0.873973 |
| 7853 | m.7853G>A | *MT-CO2* | G>A | 1148 | 0.60 | 1.00 | 0.551452 | 0.905717 |
| 11914 | m.11914G>A | *MT-ND4* | G>A | 1142 | -0.80 | 1.43 | 0.576022 | 0.905717 |
| 16086 | m.16089T>C | *D-loop* | T>C | 1148 | 1.28 | 2.35 | 0.586614 | 0.905717 |
| 12811 | m.12811T>C | *MT-ND5* | T>C | 1119 | 0.61 | 1.13 | 0.586687 | 0.905717 |
| 8964 | m.8964C>T | *MT-ATP6* | C>T | 1151 | 1.51 | 2.91 | 0.603811 | 0.905717 |
| 6680 | m.6680T>C | *MT-CO1* | T>C | 1148 | 0.52 | 1.12 | 0.639640 | 0.926375 |
| 8020 | m.8020G>A | *MT-CO2* | G>A | 1149 | 0.78 | 2.14 | 0.714468 | 0.939966 |
| 215 | m.215A>G | *D-loop* | A>G | 1142 | 1.03 | 3.12 | 0.740564 | 0.939966 |
| 14178 | m.14178T>C | *MT-ND6* | T>C | 1151 | 0.87 | 3.01 | 0.773485 | 0.939966 |
| 16327 | m.16327C>T | *D-loop* | C>T | 1151 | 0.47 | 1.85 | 0.801241 | 0.939966 |
| 7684 | m.7684T>C | *MT-CO2* | T>C | 1151 | 0.26 | 1.05 | 0.805768 | 0.939966 |
| 5108 | m.5108T>C | *MT-ND2* | T>C | 1151 | -0.46 | 1.95 | 0.812769 | 0.939966 |
| 1438 | m.1438G>A | *MT-RNR1* | G>A | 1106 | 0.34 | 2.02 | 0.865589 | 0.939966 |
| 11215 | m.11215C>T | *MT-ND4* | C>T | 1151 | 0.37 | 2.30 | 0.873716 | 0.939966 |
| 3206 | m.3206C>T | *MT-RNR2* | C>T | 1143 | -0.23 | 1.89 | 0.901237 | 0.939966 |
| 3010 | m.3010G>A | *MT-RNR2* | G>A | 1149 | -0.13 | 1.06 | 0.905134 | 0.939966 |
| 14979 | m.14979T>C | *CYTB* | T>C | 1134 | -0.20 | 1.96 | 0.917133 | 0.939966 |
| 13263 | m.13263A>G | *MT-ND5* | A>G | 1150 | 0.18 | 1.87 | 0.922906 | 0.939966 |
| 12630 | m.12630G>A | *MT-ND5* | G>A | 1151 | -0.18 | 2.40 | 0.939966 | 0.939966 |
| N = number of tests; SE = standard error; FDR = false discovery rate. | | | | | | | | |

**Table S6. Conditions for tandem mass spectrometry analysis of lipid species.**

| **Lipid Class** | **Parent Ion** | **Fragmentation** | **Number of features** | **Internal Standard** | **Internal standard (pmol)** | **Collision Energy (V)** |
| --- | --- | --- | --- | --- | --- | --- |
| Dihydroceramide (Cer(d18:0)) | [M+H]^+^ | NL, 18 Da | 4 | Cer(d18:1/17:0) | 10 | 40 |
| Ceramide (Cer(d18:1)) | [M+H]^+^ | PI, m/z 264.3 | 6 | Cer(d18:1/17:0) | 10 | 40 |
| Ceramide (Cer(t18:0)) | [M+H]^+^ | PI, m/z 264.3 | 4 | Cer(d18:1/17:0) | 10 | 40 |
| Ceramide (Cer(m18:1)) | [M+H]^+^ | PI, m/z 264.3 | 10 | Cer(d18:1/17:0) | 10 | 40 |
| Ceramide-1-Phosphate (CerP) | [M+H]^+^ | PI, m/z 264.3 | 10 | Cer(d18:1/17:0) | 10 | 40 |
| Phosphatidylcholine (PC) | [M+H]^+^ | PI, m/z 184.1 | 86 | PC(13:0/13:0) | 10 | 30 |
| Alkylphosphatidylcholine (PC(O)) | [M+H]^+^ | PI, m/z 184.1 | 47 | PC(13:0/13:0) | 10 | 30 |
| Lysophosphatidylcholine (LPC) | [M+H]^+^ | PI, m/z 184.1 | 19 | LPC(12:0) | 10 | 30 |
| Lysoalkylphosphatidylcholine (LPC(O)) | [M+H]^+^ | PI, m/z 184.1 | 14 | LPC(12:0) | 10 | 30 |
| Phosphatidylethanolamine (PE) | [M+H]^+^ | NL, 141 Da | 40 | PE(12:0/12:0) | 10 | 30 |
| Alkenylphosphatidylethanolamine (PE(P)) | [M+H]^+^ | NL, 141 Da | 35 | PE(12:0/12:0) | 10 | 30 |
| Lysophosphatidylethanolamine (LPE) | [M+H]^+^ | NL, 141 Da | 19 | LPE(14:0) | 10 | 30 |
| AlkenylLysophosphatidylethanolamine (LPE(P)) | [M+H]^+^ | NL, 141 Da | 2 | LPE(14:0) | 10 | 30 |
| Phosphatidylglycerol (PG) | [M+H]^+^ | acyl specific | 1 | PG(12:0/12:0) | 10 | 30 |
| Cholesterol ester (CE) | [M+NH_4_]^+^ | PI, m/z 369.3 | 8 | CE(17:0) | 100 | 20 |
| Monoacylglycerol (MG) | [M+NH_4_]^+^ | NL, 91 Da | 4 | MG(17:0) | 10 | 20 |
| Diacylglycerol (DG) | [M+NH_4_]^+^ | NL, NH_3_ + fatty acid | 40 | DG(15:0/15:0) | 10 | 30 |
| Triacylglycerol (TG) | [M+NH_4_]^+^ | NL, NH_3_ + fatty acid | 296 | TG(17:0/17:0/17:0) | 10 | 30 |
